# Adaptation of Droplet Digital PCR-Based HIV Transcription Profiling to Digital PCR and Association of HIV Transcription and Total or Intact HIV DNA

**DOI:** 10.3390/v15071606

**Published:** 2023-07-22

**Authors:** Carolin Tumpach, Ajantha Rhodes, Youry Kim, Jesslyn Ong, Haoming Liu, Doris Chibo, Julian Druce, Deborah Williamson, Rebecca Hoh, Steven G. Deeks, Steven A. Yukl, Michael Roche, Sharon R. Lewin, Sushama Telwatte

**Affiliations:** 1 Department of Infectious Diseases, The University of Melbourne at the Peter Doherty Institute for Infection and Immunity, Melbourne 3000, Australia; 2 Victorian Infectious Diseases Reference Laboratory, The Royal Melbourne Hospital at the Peter Doherty Institute for Infection and Immunity, Melbourne 3000, Australia; 3 Walter and Eliza Hall Institute, Melbourne 3052, Australia; 4 Department of Medicine, University of California San Francisco (UCSF), San Francisco, CA 94143, USA; 5 San Francisco Veteran Affairs Medical Center, San Francisco, CA 94121, USA; 6 Infectious and Inflammatory Diseases Theme, School of Health and Biomedical Sciences, RMIT University, Melbourne 3000, Australia; 7 Department of Infectious Diseases, Alfred Hospital and Monash University, Melbourne 3004, Australia

**Keywords:** droplet digital PCR, ddPCR, digital PCR, dPCR, QIAcuity, Bio-Rad, HIV transcription, HIV RNA, intact HIV DNA, reservoir

## Abstract

In most people living with HIV (PLWH) on effective antiretroviral therapy (ART), cell-associated viral transcripts are readily detectable in CD4+ T cells despite the absence of viremia. Quantification of HIV RNA species provides insights into the transcriptional activity of proviruses that persist in cells and tissues throughout the body during ART (‘HIV reservoir’). One such technique for HIV RNA quantitation, ‘HIV transcription profiling’, developed in the Yukl laboratory, measures a series of HIV RNA species using droplet digital PCR. To take advantage of advances in digital (d)PCR, we adapted the ‘HIV transcription profiling’ technique to Qiagen’s dPCR platform (QIAcuity) and compared its performance to droplet digital (dd)PCR (Bio-Rad QX200 system). Using RNA standards, the two technologies were tested in parallel and assessed for multiple parameters including sensitivity, specificity, linearity, and intra- and inter-assay variability. The newly validated dPCR assays were then applied to samples from PLWH to determine HIV transcriptional activity relative to HIV reservoir size. We report that HIV transcriptional profiling was readily adapted to dPCR and assays performed similarly to ddPCR, with no differences in assay characteristics. We applied these assays in a cohort of 23 PLWH and found that HIV reservoir size, based on genetically intact proviral DNA, does not predict HIV transcriptional activity. In contrast, levels of total DNA correlated with levels of most HIV transcripts (initiated, proximally and distally elongated, unspliced, and completed, but not multiply spliced), suggesting that a considerable proportion of HIV transcripts likely originate from defective proviruses. These findings may have implications for measuring and assessing curative strategies and clinical trial outcomes.

## 1. Introduction

HIV transcription profiling, which uses a panel of six droplet digital PCR (ddPCR)-based primer/probe sets to simultaneously quantify mechanistically important HIV transcripts [1], is an established technique used to quantitate HIV transcriptional activity in samples. In this study, we aimed to determine the suitability of a digital (d)PCR platform for high-throughput HIV transcription profiling measurements. Accordingly, ddPCR (Bio-Rad) and dPCR (Qiagen’s QIAcuity) platforms were directly compared using HIV transcription profiling with varied inputs of in vitro transcribed RNA and supernatant virus RNA under near-identical reaction conditions.

Both dPCR and ddPCR technologies feature a method to partition reactions (either into discrete droplets or into nanowells), acquire data at the reaction end point, and yield independent, absolute quantification without standard curves, thereby improving precision and reproducibility over qPCR [2]. These technologies may also be less influenced by sequence mismatches with the target or the presence of sample contaminants that can partially inhibit Taq polymerase and/or primer annealing [3,4].

Qiagen’s QIAcuity instrument offers the advantage of performing partitioning, thermocycling, and imaging as a single, fully automated instrument, which considerably reduces hands-on time, with shorter time to results. It additionally features advanced multiplexing capabilities, which will enable future optimization of other emerging techniques to assess HIV transcription and HIV DNA harbored by infected cells.

## 2. Materials and Methods

The objectives of this study were to validate HIV transcription profiling assays using a dPCR platform, directly compare the data quality to the ddPCR platform, and subsequently assess levels of HIV transcription in individuals living with HIV on suppressive therapy relative to their levels of infected cells (reservoir size). As such, great care was taken to ensure that all experimental procedures minimized differences arising from confounding factors (i.e., dPCR and ddPCR experiments were performed in parallel using the same cDNA aliquots and primer/probe aliquots). Notwithstanding, given the differences in platform, different probe mastermixes (Bio-Rad’s ddPCR Supermix (no dUTP) vs. Qiagen’s QIAcuity Probe MasterMix) and data acquisition platforms (ddPCR vs. dPCR) were required.

### 2.1. Ethics Statement

In total, 23 people living with HIV (PWLH) on antiretroviral therapy (ART) were recruited for leukapheresis sampling at The Alfred and Avenue Hospitals in Melbourne, Australia (n = 10), and University of California San Francisco (UCSF), USA (n = 13). Samples from individuals with documented HIV infection aged 18 years or older and receiving combination ART with plasma HIV RNA < 50 copies/mL for at least 2 years were studied. Use of samples was approved by Human Research Ethics committees at the Alfred Hospital in Melbourne, the University of Melbourne, and the Institutional Review Board at UCSF.

### 2.2. Nucleic Acid Extractions

RNA and DNA extractions from CD4+ T cells isolated from cryopreserved PBMC were performed by either 1 mL TRI Reagent containing 2.5 μL polyacryl carrier (both from Molecular Research Center, Cincinnati, OH, USA) as previously described [5] or AllPrep DNA/RNA Mini kit (Qiagen, Hilden, Germany) as per manufacturer guidelines.

### 2.3. Preparation of HIV-1 RNA Standards

Two HIV-1 RNA standards were prepared for this study as previously described: (1) a ‘virion’ standard comprising RNA extracted from clarified and multi-nuclease treated NL4.3 viral supernatant, and (2) an in vitro transcribed ‘IVT’ standard using a custom-designed plasmid template that encodes TAR, LongLTR, Nef, PolyA, and Tat-Rev [1]. This IVT standard does not include Pol, so the virion standard was used for all Pol testing. Aliquots of undiluted virion standard RNA were quantified in triplicate using a HIV-1 viral load assay (Roche cobas 6800 analyzer), and the IVT standard was quantified using qPCR and by RNA mass using a Nanodrop One spectrophotometer to determine copy numbers by methods independent of dPCR or ddPCR. The standards were accordingly diluted to working concentrations and stored at −80 °C prior to use.

### 2.4. Reverse Transcription for ‘HIV Transcription Profiling’ Assays

Dilutions of the supernatant virus standard or in vitro transcribed standard were added to reverse transcription reactions to achieve expected inputs of 1 to 10,000 copies per 5 μL cDNA. Reverse transcription (RT) reactions were performed in 50 μL reaction mixture containing 5 μL of 10× SuperScript III buffer (Invitrogen), 5 μL of 50 mM MgCl_2_, 2.5 μL of random hexamers (50 ng/μL; Invitrogen), 2.5 μL of 50 μM poly-dT15, 2.5 μL of 10 mM deoxynucleoside triphosphates (dNTPs), 1.25 μL of RNAseOUT (40 U/μL; Invitrogen), and 2.5 μL of SuperScript III RT (200 U/μL; Invitrogen) [1]. The resultant cDNA was split evenly across triplicate wells for ddPCR and dPCR assayed for HIV transcription profiling assays: TAR, Long LTR, Pol, Nef, PolyA, and Tat-Rev [5].

### 2.5. Droplet Digital PCR

Droplet digital PCR reactions consisted of 20 μL mixture per well containing 10 μL of ddPCR Probe Supermix (no deoxyuridine triphosphate, Bio-Rad, Hercules, CA, USA), 900 nM of primers, 250 nM of probe, and 5 μL of cDNA generated from either HIV-1 virion RNA or the ‘IVT’ standards. The ddPCR reactions were incorporated into droplets using the QX100 Droplet Generator (Bio-Rad). Nucleic acids were amplified with the following cycling conditions: 10 min at 95 °C, 45 cycles of 30 s at 95 °C and 59 °C for 60 s, and a final droplet cure step of 10 min at 98 °C using a Vapo.Protect Mastercycler^®^ (Eppendorf, Hamburg, Germany). Droplets were read and analyzed using Bio-Rad QX200 system and QuantaSoft software (version 1.7.4.0917) in ‘absolute quantification’ mode. Only wells containing ≥11,000 droplets were accepted for further analysis. No-RT controls (containing ≥10,000 cp HIV RNA) and no-template controls (NTCs) were included in every independent experiment.

### 2.6. QIAcuity dPCR

QIAcuity dPCR reactions were conducted in parallel with ddPCR reactions by the same operator using cDNA generated from common RT reactions. All assays were validated using the QIAcuity Four 5-plex digital PCR System (Qiagen, Hilden, Germany).

Digital PCR reactions consisted of 40 μL reaction mixture per well containing 10 µL QIAcuity 4× Probe PCR master mix (Qiagen), 900 nM of primers, 250 nM of probe, PCR-grade water (Thermo Fisher Scientific, Waltham, MA, USA), and DNA template. Assembled reactions were transferred into QIAcuity 26k 24-well Nanoplates (Qiagen) for partitioning using the Qiagen Standard Priming Profile, and nucleic acids were amplified under the following conditions: enzyme activation for 2 min at 95 °C and 45 cycles of 15 s at 95 °C and 30 s at 59 °C. Partitions were imaged with 400 ms (FAM)/300 ms (VIC) exposure time, with gain set to 6 for both target channels. The QIAcuity Software Suite (Qiagen, version 2.1.7) was used to determine sample thresholds using positive, negative, and no-template control wells (NTCs) with the manual global threshold approach that is based on the amplitude signal observed in negative control samples [6].

### 2.7. Calculations for Limit of Blank (LoB), Limit of Detection (LoD), Limit of Quantitation (LoQ), and Intra- and Inter-Assay Variability (%CV)

To compare analytic assay metrics across the dPCR and ddPCR platforms, we calculated the Limit of Blank (LoB), Limit of Detection (LoD), and Limit of Quantitation (LoQ) for each of the HIV transcription profiling assays.

The Limit of Blank (LoB), defined as the highest concentration that can be found in no-template controls (NTCs), was determined using the following formula [7,8]:

LoB = mean_NTC_ + 3 × SD_NTC_

where SD = standard deviation.

The Limit of Quantification (LoQ; the lowest concentration of target cDNA that can be detected with sufficient confidence) was approximated using a 10-fold dilution series ranging from 10,000 copies to 1 copy to determine the lowest copy input that was reliably detected in ≥90% of replicates (‘lowconcentrationsample’) (21–30 replicates/assay).

The Limit of Detection (LoD) was then calculated from LoB using the standard deviation from the lowest detectable concentration of positive control using the following formula:

LoD = LoB + 3(SD_lowconcentrationsample_)

The LoD represents the lowest copy input that is likely to be reliably distinguished from the LoB and is feasibly detected [7].

To determine the reproducibility of each primer/probe set in both platforms, the intra- and inter-assay variability was calculated as follows.

Intra-assay coefficient of variation (%CV) = average(SD of triplicate mean ÷ triplicate mean × 100)(1)

Inter-assay %CV = SD of plate means ÷ mean of plate means × 100(2)

Each primer/probe set was tested at inputs ranging from 10,000 copies to 1 copy in at least triplicate within a plate.

### 2.8. Intact Proviral DNA Assay

Levels of genetically intact, 3′-defective, and 5′-defective proviral DNA were assessed by the Intact Proviral DNA Assay (IPDA), adapted to the QIAcuity dPCR system [9], using published primer/probe sets [10] along with the ‘Secondary env’ primer/probe set [11] applied to samples for which the env primer/probe set failed due to sequence mismatches: Secondary env Forward Primer (ACTATGGGCGCAGCGTC), Secondary env Reverse Primer (CCCCAGACTGTGAGTTGCA), and Secondary env Probe (VIC—CTGGCCTGTACCGTCAG—MGB).

### 2.9. HIV Transcription Profiling of CD4+ T Cells from PLWH

CD4+ T cells were isolated from cryopreserved peripheral blood mononuclear cells (PBMC; 10^7^ cells, using the EasySep Human CD4+ T cell Isolation Kit (StemCell Technolgies, Vancouver, BC, Canada)). RNA and DNA concentrations and quality were assessed using the Nanodrop One spectrophotometer (Thermo Fisher Scientific, Waltham, MA, USA). Up to 1 μg of total RNA was used for a polyadenylation–reverse transcription–ddPCR assay for the TAR region, and up to 5 μg of RNA was used for a separate 50 μL common RT reaction, from which aliquots of cDNA (5 μL/well) were used in dPCR assays for other sequence regions, including R-U5/pre-Gag (‘Long LTR’), unspliced (‘Pol’), Nef, U3-R-polyA (‘Poly A’), and multiply spliced Tat-Rev (‘Tat-Rev’) regions [1]. RT reactions were performed as described in Section 2.4, followed by digital PCR using the QIAcuity as described in Section 2.6.

### 2.10. Statistical Analyses

Statistical analyses were performed using GraphPad Prism (version 9.5.1). The correlation between dPCR and ddPCR for a given assay was determined using a Spearman rank-order correlation. Differences between dPCR and ddPCR quantitation for each assay were calculated using Wilcoxon matched-pairs signed rank test. Assay linearity and efficiencies were determined using linear regression. Nonparametric Spearman rank-order correlation was computed to assess the relationship between intact HIV DNA/total HIV DNA/years of suppression under ART and each HIV transcript.

## 3. Results

### 3.1. Assay Optimization for dPCR

We tested a range of probe concentrations to determine the optimal conditions required for dPCR. As expected, the amplitudes of positive and negative partitions shifted in a concentration-dependent manner. The highest concentration of probe (500 nM) resulted in saturation at an exposure of 400 ms/gain of 6, despite improved signal-to-noise ratio (Figure S1). At all concentrations, we observed less ‘rain’ using the QIAcuity compared to ddPCR, yet the overall quantification across platforms was not significantly different. Consequently, based on these data, we opted to maintain the same primer/probe concentrations for dPCR (250 nM) as used for ddPCR.

### 3.2. Fluorescence Signal-to-Noise Ratio and Primer/Probe Efficiency

**Figure 1:**
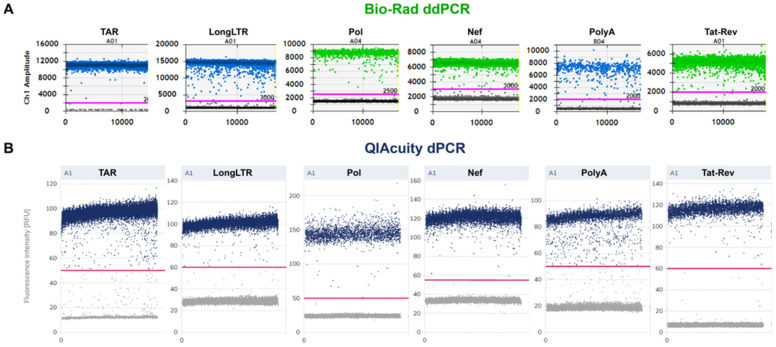
Signal-to-noise comparisons for ddPCR and dPCR. One-dimensional plots for (**A**) Bio-Rad ddPCR and (**B**) QIAcuity dPCR, showing inputs of 10,000 copies of respective RNA standard. Scale is shown to the right of each plot. Threshold gating is indicated by horizontal lines in pink (**A**) or red (**B**). Bio-Rad’s Quantasoft program discriminates plots in its two channels by color: probes in FAM are indicated in blue, whereas probes in VIC are shown in green. Negative droplets/partitions are shown in grey.

Primer/probe efficiencies were assessed by separation between negative and positive droplets/partitions [2] (Figure 1). As expected, the signal-to-noise ratios (SNR) for each assay tested tended to vary across platforms using the same primer/probe concentrations. However, for all assays except Nef, across both platforms, the SNR was >4 (range: 4.2–21.9), which is an in-house benchmark for optimal discrimination of positive and negative populations (partitions or droplets). For Nef, the SNR was identical across the two platforms (SNR = 3.73), which suggests that the platform used had no bearing on assay performance under the same conditions. Taken together, the SNR data support good primer specificity and reaction efficiency across all tested primer/probe sets.

### 3.3. Assessment of Linear Dynamic Range and Precision of ddPCR and qPCR Technologies for Low Target Concentration

We determined the efficiency, linearity, and sensitivity for each primer/probe set using terminal dilutions of HIV-1 RNA standards. Briefly, cDNA was added to ddPCR and dPCR wells at expected inputs of 1–10,000 copies/well in triplicate (10,000, 1000, 100, and 1 copy) or six replicates (10 copies).

‘No RT’ controls were routinely negative, with the exception of rarely detected single droplets for TAR and Pol (overall rate across all replicates: 0.06 and 0.05, respectively) in both dPCR and ddPCR platforms. These were often detected as artifactual high fluorescence droplets (‘stars’) sometimes observable in low target concentration samples [8]. Similarly, NTCs were also routinely negative, with rare single droplets/partitions detected for TAR, LongLTR, Pol, and Tat-Rev, which were used to calculate the LoB for each respective assay across both platforms.

**Figure 2:**
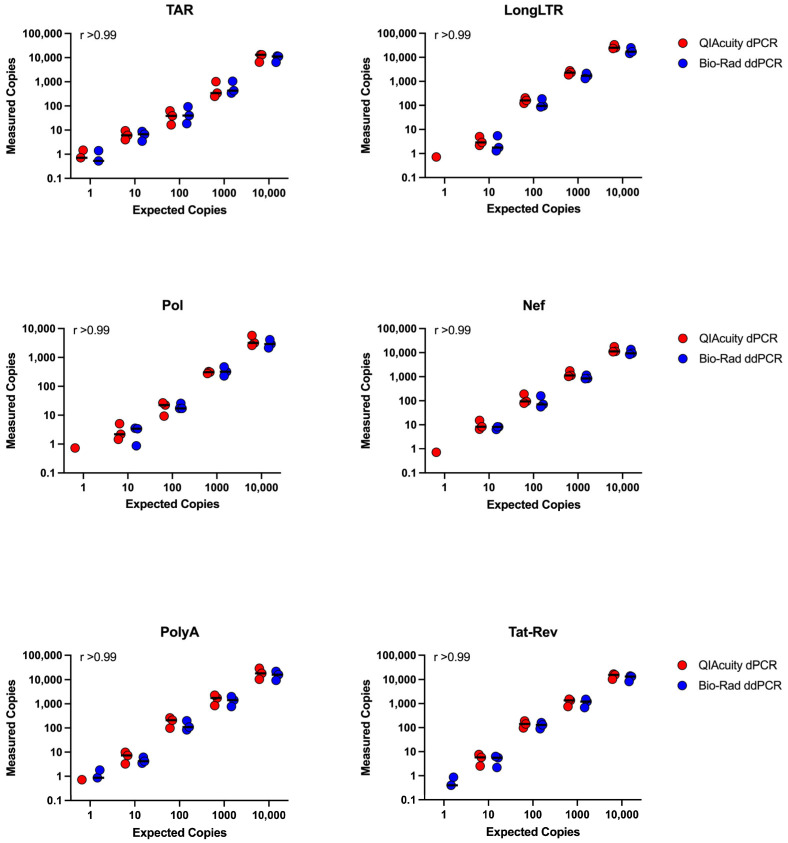
Efficiency and linearity of ddPCR assays for HIV targets determined in parallel dPCR and ddPCR reactions using same cDNA inputs. r = Spearman’s rank correlation coefficient, *p* = 0.017 for all primer/probe sets. Data for primer/probe sets for TAR, LongLTR, Pol, Nef, PolyA, and Tat-Rev are shown (n = 3 independent experiments).

All assays showed linear quantification (i.e., no significant deviation from linearity), with R^2^ > 0.99 for all primer/probe sets regardless of platform (dPCR or ddPCR) and a linear dynamic range of ≥4 orders of magnitude (Figure 2).

For both dPCR and ddPCR, the LoQ was likely <10 copies/well for TAR, LongLTR, Nef, PolyA, and Tat-Rev (i.e., 10 HIV copies were reliably detected at least 90% of the time [8] (Table 1)). For Pol, 10 HIV copies were reliably detected at least 81% of the time with both platforms, suggesting the LoQ was likely to be close to 10 copies, but the precise LoQ for Pol was not determined in this study. Importantly, there was no difference in estimated LoQ between ddPCR and dPCR for any assay tested.

Across all assays, the average LoB was 0.568 copies/well for dPCR (range: 0–1.917) and 0.338 copies/well for ddPCR (range: 0–0.737) and the average LoD was 3.198 copies/well for dPCR (range: 1.247–7.619) and 2.33 copies/well for ddPCR (range: 0.814–4.298; Table 1), indicating that Qiagen’s QIAcuity exhibits similar sensitivity across a range of metrics to Bio-Rad’s QX200.

### 3.4. Intra- and Inter-Assay Variability

Next, we assessed the reproducibility of each primer/probe set by determining the intra- and inter-assay variability (% coefficient of variation (%CV)). We found the intra-assay %CV for dPCR was below the acceptable threshold of 10 [7,12] for all primer/probe sets tests (range: 3.38–9.93; Table 1) and did not differ significantly from that of ddPCR (range: 3.3–13.0).

At high copy inputs between 1000 and 10,000, the inter-assay %CV was similarly low (dPCR range: 2.26–8.33; ddPCR range: 1.59–4.67). As expected, we observed greater variability at lower copy inputs of 10 to 100 copies (dPCR range: 8.72–46.16; ddPCR range: 8.54–33.59). Nonetheless, for dPCR in particular, four of the six primer/probe sets were close to the 25% inter-assay threshold [12].

Overall, all primer/probe sets exhibited similar reproducibility across the dPCR and ddPCR platforms.

### 3.5. Concordance between ddPCR and dPCR Platforms

We determined the degree of association of quantitation between QIAcuity dPCR and QX200 ddPCR platforms for all assays validated (TAR, LongLTR, Pol, Nef, PolyA, and Tat-Rev). The Spearman’s rank correlation coefficient for each primer/probe set was r > 0.99 (*p* = 0.017 for all; Figure 2), highlighting the very high concordance between data obtained from both platforms.

To assess and compare the absolute quantification across the two platforms, the ratios of ‘measured copies’ to ‘expected copies’ were calculated for every input (10,000, 1000, 100, and 10 copies, ±1 copy input where detectable) for each assay (TAR, LongLTR, Pol, Nef, PolyA, and Tat-Rev) for both dPCR and ddPCR. The average difference in these ratios across platforms for each assay was calculated as a percentage to determine the performance of the QIAcuity relative to the QX200 in detecting identical inputs derived from common RT reactions (Table 2). We found no consistent difference in quantification across the two platforms (*p* > 0.25 for all).

### 3.6. Levels of HIV Transcription in People Living with HIV Are Not Influenced by Size of the Intact Reservoir, Duration of Suppressive ART, or HLA Allele Carriage

**Figure 3:**
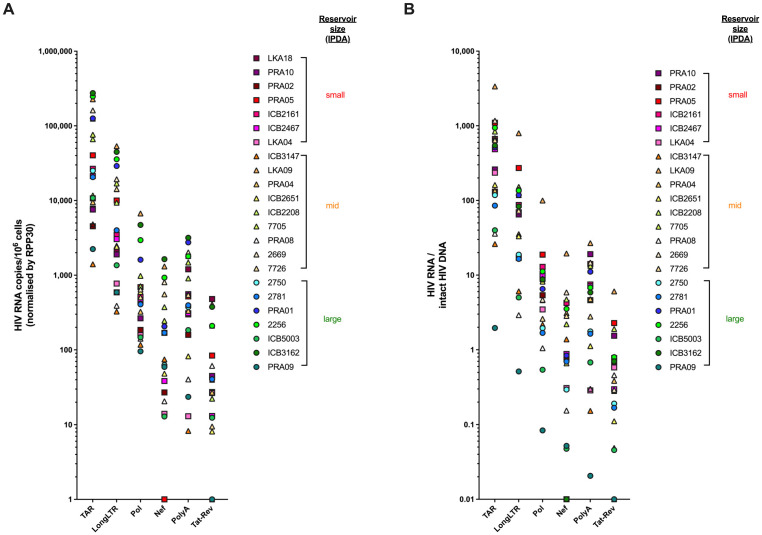
Intact reservoir size does not correspond to levels of HIV transcription. Expression of each HIV transcript species was measured by dPCR and expressed as (**A**) HIV RNA copies per million cells (normalized by RPP30) and (**B**) HIV RNA copies per intact provirus. LKA18 is not shown, as no intact HIV DNA was detected in this participant. Samples were stratified by reservoir size (‘small’ (square symbols), ’mid’ (triangle symbols), and ‘large’ (circle symbols)), as estimated by the levels of intact proviral DNA using the ‘Intact Proviral DNA Assay’.

The dPCR-validated transcription profiling assays were applied to samples from 23 people living with HIV (PLWH) (Figure 3). The study participants were HIV-infected adults on suppressive ART from two cohorts (median age = 55; median CD4 count = 474 cells/mm^3^; median years of suppression = 11; Table 3). For each participant, we first determined the levels of genetically intact proviral DNA in addition to 3′-defective and 5′-defective DNA, employing a widely adopted method (intact proviral DNA assay (IPDA) [10]) using DNA extracted from the same samples. The participants within the cohort were stratified by ‘intact’ reservoir size based on the median and interquartile range of the IPDA data: ‘small’ = <50 copies; ‘mid’ = ≥50–200 copies; and ‘large’ = >200 copies of intact HIV DNA/million CD4+ T cells. The corresponding levels of total HIV DNA (sum of intact, 3′-defective, and 5′-defective HIV DNA) were a median of 12.5-fold higher than intact HIV DNA (range: 1.49–67.25-fold; Figure S2), in line with expectations.

We next measured the levels of TAR, LongLTR, Pol, Nef, PolyA and Tat-Rev in isolated CD4+ T cells for each participant in our cohort by RT-dPCR and normalized them by multiple measures (housekeeping gene RPP30 (Figure 3A), RNA mass, and DNA mass (Figure S3)). The average levels of each HIV transcript per intact provirus were determined using the ratio of each HIV transcript to intact HIV DNA (Figure 3B). In line with our previous observations [1,5], we observed a step-wise decline in levels of HIV RNA regardless of the method of normalization: total (TAR) > elongated (LongLTR) > polyadenylated > multiply spliced Tat-Rev. Further, when we normalized to the level of total HIV DNA, this step-wise decline remained consistent (Figure S3B). In our cohort, we observed some instances of lower levels of Pol and Nef relative to PolyA, which might be suggestive of deletions or deleterious mutations within those regions in some of the proviruses assumed to be ‘intact’ using the IPDA.

**Figure 4:**
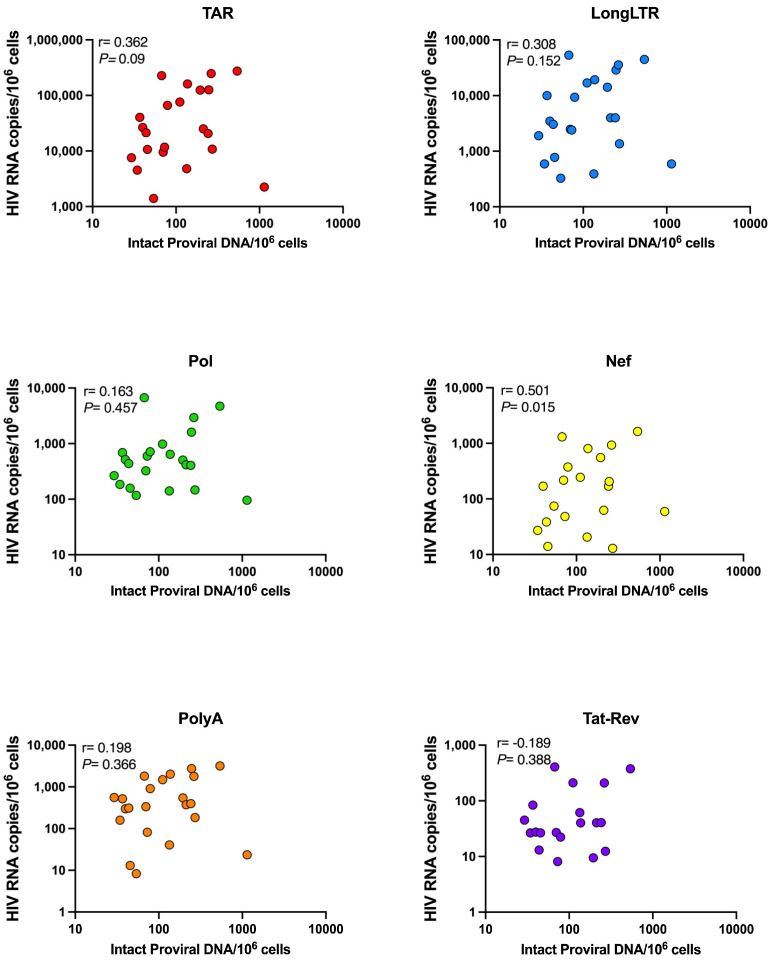
Correlation between intact reservoir size and HIV transcripts. HIV RNA copies per million CD4+ T cells normalized by RPP30 for each transcript species are shown. Spearman correlation (r) between intact proviral DNA copies and HIV transcripts. *P* = *p*-value.

Interestingly, while some individuals with a large reservoir exhibited relatively high levels of HIV transcriptional activity (for instance ICB3162, PRA01, 2256), some individuals with a ‘small’ intact reservoir, including ICB2161 and PRA05, also tended to have similarly high levels of HIV transcripts. In contrast, for participants 2781, ICB5003, and PRA09, the intact reservoir was characterized as ‘large’ (242, 271, and 1142 intact DNA copies/million cells, respectively), yet these individuals tended to show lower overall levels of HIV transcripts (Figure 3). These observations held true regardless of normalization method (Figure S3), suggesting that even small intact HIV reservoirs can exhibit relatively high transcriptional activity. We found no correlation between the levels of intact HIV DNA and TAR, LongLTR, Pol, PolyA, or Tat-Rev HIV RNA (Figure 4). We did, however, observe a correlation between intact DNA and levels of Nef RNA (Spearman r = 0.501, *p* = 0.015; Figure 4). Furthermore, we found no correlation between HIV transcript levels and years of suppression under ART (Figure S4) or HLA allele carriage (Table 3). In our small cross-sectional cohort, there was no clear association between carriage of protective and/or deleterious HLA alleles and the size of the intact reservoir or HIV transcriptional activity under ART. For instance, while PRA09 carries deleterious HLA-B*35 and exhibited a ‘large’ intact reservoir, levels of all HIV transcripts were lower than the average levels across the cohort (Figure 3). PRA02 carries the protective allele B*27, which is associated with low levels of HIV DNA in chronic HIV infection under ART [13] and indeed has both a ‘small’ intact reservoir and lower HIV transcriptional activity (Figure 3). However, PRA04 was heterozygous for HLA-B*35 and B*55, which are both markers of HIV disease progression [14], yet their intact reservoir was in the ‘mid’-range, and levels of HIV transcripts were below the average across the cohort for all HIV RNA species. In sum, there were no clear trends in HLA allele carriage and either the size of the intact reservoir or levels of HIV transcription under suppressive therapy in the study cohort.

**Figure 5:**
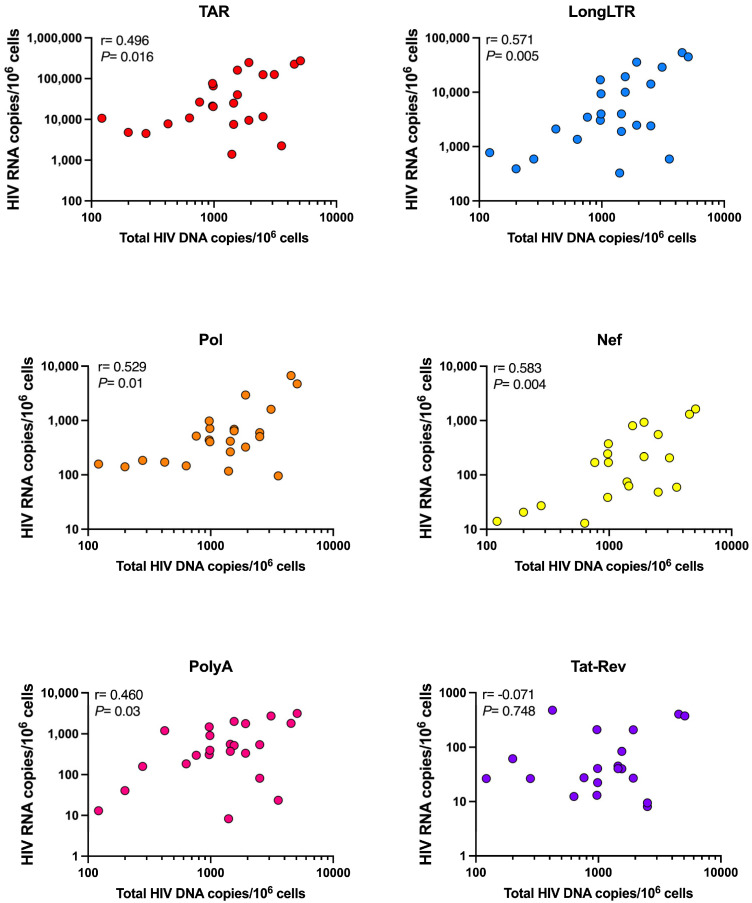
Correlation between total reservoir size and HIV transcripts. HIV RNA copies per million CD4+ T cells normalized by RPP30 for each transcript species are shown. Total DNA is presented as the sum of 5′-defective, 3′-defective, and intact HIV DNA. Spearman correlation (r) between total proviral DNA copies and HIV transcripts. *P* = *p*-value.

In contrast to these parameters, the levels of total HIV DNA (sum of 5′-defective, 3′-defective, and intact DNA) appeared to correlate with levels of all HIV RNA species except for Tat-Rev (Figure 5). We observed a positive correlation between total DNA and initiated (TAR), proximally (LongLTR) and distally (Nef) elongated, unspliced (Pol), and completed (PolyA) HIV transcripts (*p* < 0.03 for all), while there was no correlation with multiply spliced (Tat-Rev) transcripts (r = −0.071, *p* = 0.748).

Taken together, these data suggest that larger ‘intact’ reservoirs do not necessarily predict higher levels of transcriptionally active proviruses despite presumed genetic ‘intactness’.

## 4. Discussion

As the landscape of absolute quantitation by PCR evolves, adaptation of existing assay capabilities to new technology is necessary to harness those advancements.

In this study, six ddPCR-based primer/probe sets that comprise the HIV transcription profiling technique (TAR, Long LTR, Pol, Nef, PolyA, and Tat-Rev) were validated with the QIAcuity dPCR platform. We performed head-to-head experiments using the same RNA standard inputs to validate our primer/probe sets with the dPCR platform. Our validation evaluated key assay parameters including linearity, sensitivity, specificity, precision, and reproducibility in carefully controlled experiments.

Dilution series of RNA standards demonstrated that each primer/probe set displayed similar linear quantification over a dynamic range of ≥4 orders of magnitude, with R^2^ values > 0.99 for all. Our assays were highly sensitive, with the average LoD being 3.2 copies for dPCR and 2.3 for ddPCR, and also specific, as the average LoB was <1 copy irrespective of platform. We found no difference in precision across the platforms (Table 1), and intra-assay %CV values were below accepted thresholds [12]. Both platforms performed similarly for reproducibility, in that most inter-assay %CV values were within the acceptable range. The exceptions were LongLTR, Nef, and Tat-Rev, where we observed more inter-assay variability at the ‘low copy’ input, which was mostly driven by variability in the six replicates of 10-copy input routinely performed for each plate.

In head-to-head experiments, quantitation between the QIAcuity dPCR and QX200 ddPCR platforms was highly correlated (Spearman r > 0.999, *p* = 0.017 for all; Figure 2), suggesting that HIV transcription profiling performs similarly across the two platforms.

Qiagen’s dPCR platform offers less hands-on time and expanded options to multiplex in a single instrument. Among its several noted differences from Bio-Rad’s ddPCR instrument is its capture of fluorescence data. QIAcuity’s optical system employs high-power light-emitting diodes (LEDs) as the excitation source for fluorescent dyes, combined with specific excitation filters to illuminate each well. Light emitted from the fluorophores in each partition is filtered by a detection filter and imaged through an objective lens on a CMOS-camera chip [15]. Bio-Rad’s QX200 droplet reader also uses LEDs as the excitation source but instead features a multipixel photon counter to detect signal from samples. Following the droplet generation and thermal cycling processes, the QX200 Droplet Reader collects binary data for analysis as it singulates the ~20,000 nanoliter-sized droplets in each sample and then streams them in single-file past a two-color detector (multipixel photon counter) to determine which droplets contain target molecules (positive reading) and which do not (negative reading) [16,17].

Given the differences in assay format and fluorescence data acquisition, it was expected that the SNR may vary between instruments, but we observed an SNR of ~4 (which we consider to be acceptable for discrimination of positive and negative partitions/droplets) across all assays in both platforms (Figure 1).

In conclusion, the ddPCR-based HIV transcription profiling assays were easily adapted to the QIAcuity instrument, and all primer/probe sets performed equally well across numerous parameters relative to Bio-Rad’s ddPCR instrument including linearity, sensitivity, specificity, precision, and reproducibility.

By applying our dPCR-adapted assays to CD4+ T cells from PLWH and measuring the levels of genetically intact proviral DNA, we demonstrate that size of the intact reservoir alone may not directly correlate with levels of transcriptional activity. Using samples for which RNA and DNA were extracted simultaneously, we measured both the levels of intact proviral DNA and each HIV transcript species in our panel. Samples were assigned as having a ‘large’-, ‘mid’-, or ‘small’-sized reservoir based on levels of intact proviral DNA detected. We found that the spread of intact HIV DNA levels observed in participants in our study was similar to that observed in participants in the Bruner et al., 2018 [10] study in which IPDA was first applied.

Notably, the stratification of samples based on size of the ‘intact’ reservoir does not predict the level of HIV transcriptional activity, i.e., a ‘large’ intact reservoir does not necessarily correspond to higher HIV transcription (Figure 3B). We found no correlation between intact HIV DNA and any HIV transcript except for Nef (Figure 4). Interestingly, Nef is considered to be unnecessary for viral replication in both quiescent and activated T cells [18], and defects therein are often not considered in analyses assessing full-length proviral DNA [19,20]. Instead, our finding that the levels of total HIV DNA (5′-defective, 3′-defective, and intact DNA) appeared to correlate with levels of all HIV RNA species except for Tat-Rev (Figure 5) may support other data suggesting that transcriptionally active proviruses are largely defective, even those that are also translationally competent [20].

An important caveat is that we did not measure genetically intact proviruses by more comprehensive methods such as nearly full-length individual proviral sequencing (FLIP-Seq) [21,22] or matched integration site and proviral sequencing (MIP-Seq) [23], which were outside the scope of this study. These techniques can yield in-depth insight into the precise nature of genetic defects harbored by proviruses and, for MIP-Seq, insight into the matched integration sites that is not assessable by IPDA alone, so the contribution of proviruses integrated in transcriptionally silent regions to the size of the reservoir in a given participant was not assessed in this study.

Prior studies suggest that a rapid decline in intact proviruses occurs in the first 7 years of antiretroviral therapy, after which the per-year decline progresses more slowly, and that these declines in the intact reservoir occur more rapidly than for defective provirus [24]. Transcriptionally active proviruses likely exhibit higher vulnerability to elimination through host immune-mediated activity, leading to the selection of proviruses that are less transcriptionally active [25]. In our cohort, we found no correlation between the levels of HIV transcripts and the years of suppression under ART (Figure S4), but our study was limited to analysis of cross-sectional samples only.

Genetic factors such as human leukocyte antigens (HLA) are known to influence viral control pre-ART, immune escape, and disease progression [26,27], with HLA-B*27 and B*57 alleles associated with slower disease progression and long-term control of HIV-1 replication [28,29,30,31], while B*46 [32], B*35, and B*53 [33,34,35] alleles are associated with faster disease progression. Although we observed no trends in protective/deleterious HLA-B allele carriage and size of the intact reservoir and/or HIV transcriptional activity under ART in this study, our cohort was too small to comprehensively assess the association of these parameters.

Our observation of lower Pol and Nef RNA levels relative to PolyA (a measure of polyadenylated/completed transcripts) in some participants may be indicative of deletions in these regions, which are known to occur frequently [19,21,36]. We have previously shown that intact HIV RNA is only transcribed by a small fraction (median of 2.2%) of intact proviruses, and we found a vast excess of 3′-defective HIV RNA compared to 5′-defective HIV RNA that was not observed for HIV DNA [37], which points to transcriptional blocks and/or high rates of HIV RNA turnover. In this study, transcription profiling analysis of our cohort suggests that there may also be potential deletions in integrated proviruses not detected using the IPDA. The clinical significance of defective yet inducible proviruses remains to be understood [38], but these findings underscore the contribution of defective proviruses to the transcriptionally active reservoir, which is an important consideration for measuring and assessing curative strategies and clinical trial outcomes. Taken together, these findings add additional insight into the transcriptional activity of the genetically intact and whole HIV reservoir under suppressive therapy.

## Figures and Tables

**Table 1:** Intra- and inter-assay variability of all primer/probe sets across dPCR and ddPCR platforms.

Assay (Primer/Probe Set)	Intra-Assay ^1^ %CV		Inter-Assay ^2^ %CV (High Copy)		Inter-Assay %CV (Low Copy)		Frequency of 10 Copies Detected (%) ^3^	
	dPCR	ddPCR	dPCR	ddPCR	dPCR	ddPCR	dPCR	ddPCR
TAR	8.59	7.7	8.33	4.24	22.67	18.56	95	95
LongLTR	4.97	4.1	4.4	3.68	39.2	33.01	90	90
Pol	9.93	13.0	4.53	4.27	26.59	9.56	81	83
Nef	3.38	4.32	2.97	4.67	18.63	28.14	100	100
PolyA	7.02	3.3	2.26	1.59	8.72	8.54	100	100
Tat-Rev	4.86	5.30	2.88	3.34	46.16	33.59	90	94

^1^ Intra-assay variation was calculated for inputs of 100, 1000, and 10,000 copies. ^2^ Inter-assay variation calculated for high copies = 1000 and 10,000, low copies = 10 and 100 copies. ^3^ ‘Frequency of 10 copies detected’ is expressed as a percentage of all replicates analyzed.

**Table 2:** Difference in measured vs. expected HIV copies across dPCR and ddPCR platforms.

Assay	RNA Standard ^2^	dPCR	ddPCR	
		Average Measured/Expected Copies	Average Measured/Expected Copies	Difference (%)
TAR	IVT	0.78	0.71	6.6
LongLTR	IVT	1.44	1.20	24.29
Pol ^1^	VIR	0.273	0.259	1.4
Nef	IVT	1.13	0.79	34.43
PolyA	IVT	1.51	1.25	26.51
Tat-Rev	IVT	0.8	0.76	3.95

^1^ Quantification for Pol was undertaken using the ‘virion’ standard, for which the levels of Pol are expected to be lower than LongLTR, upon which the copy numbers were originally estimated. ^2^ IVT = RNA standard in vitro transcribed from synthetic plasmid, VIR = virion standard prepared from HIV-1 cultured supernatant.

**Table 3:** Demographic data for study participants.

Participant ID	Age (Years)	Sex	Race	HIV Diagnosis	CD4+ Count (Cells/μL)	CD4 (%)	CD8+ Count (Cells/μL)	CD8 (%)	Nadir CD4+ Count (Cells/μL)	ART Regimen ^1^	VL (Copies/mL)	Peak VL (Copies/mL)	Duration HIV RNA < 50 Copies (Years)	HLA-B Alleles ^3^
ICB2161	69	M	Caucasian	1985	800	42	647	34	98	3TC, DRV, RTV, DTG	<40	80,410	7	14:01:01G + 27:05:02G
ICB2208	66	M	Caucasian	1984	466	31	546	36	54	FTC/TAF, DRV/COBI	<40	50,000	9.8	07:02:01G + 57:01:01G
ICB2467	46	M	Hispanic/Latino	2006	429	43	316	32	324	RPV/TAF/FTC	<40	47,100	10.4	39:05:01G + 48:01:01G
ICB2651	52	M	Caucasian	2001	655	37	681	39	275	ABC/DTG/3TC	<40	45,069	14	14:02:01G + 14:02:01G
ICB3147	61	M	Hispanic/Latino	1993	837	44	522	27	4	ABC/DTG/3TC	<40	119,870	11	44:02:01G + 52:01:01G
ICB3162	56	M	Caucasian	1987	586	37	471	30	200	DRV, RTV, ABC/DTG/3TC	<40	171,000	11.5	07:02:01G + 51:09:01G
ICB5003	47	M	Caucasian	1993	279	25	385	35	56	ATV, ABC/DTG/3TC	<40	171,000	6.7	39:01:01G + 52:01:01G
LKA04	56	M	Caucasian	1977	769	35	530	24	230	ABC/3TC/DTG	<20	NA	28	18:01:01G + 51:01:01G
LKA09	51	M	Caucasian	1997	372	27	771	56	72	TDF/3TC, ATV, RAL	<20	199,100	13.49	07:02:01G + 50:01:01G
LKA18	60	M	Caucasian	1993	312	19	312	34	2	TAF/FTC	<20	NA	12	41:02:01G + 44:15:01G
PRA01	64	M	Caucasian	1985	403	24	1061	63	10	ATV, TDF/FTC	<20	148,430	14.1	08:01:01G + 44:02:01G
PRA02	48	M	Caucasian	2006	1460	47	793	26	698	ABC/3TC, EFV	<20	NA	12	27:05:02G + 39:01:01G
PRA04	55	M	Caucasian	1996	1036	40	1069	42	266	ATV, TDF/FTC	<20	100,000	11.1	35:01:01G + 55:01:01G
PRA05	49	M	Caucasian	2003	388	28	717	51	168	TAF/FTC, MVC	<20	146,000	12	08:01:01G + 08:01:01G
PRA08	38	M	Other (PNG)	2006	281	25	328	30	168	EVG/TAF/FTC/COBI	<20	63,300	13	40:01:01G + 40:02:01G
PRA09	49	M	Caucasian	2010	474	25	1085	56	42	EVG/TAF/FTC/COBI	<20	211,930	7	35:01:01G + 51:01:01G
PRA10	48	M	Caucasian	2000	484	28	895	52	411	TAF, FTC, RPV	<20	N/A ^2^	N/A	40:01:01G + 50:01:01G
2256	62	M	Caucasian	1985	310	25	550	45	86	RPV/TAF/FTC, TCV	<40	29,900	13.2	15:02 + 40:01
2669	59	M	Caucasian	1989	420	24	672	38	180	ABC/TCV/3TC	<40	900,000	8.39	14:02 + 44:03
2750	56	M	Caucasian	2005	474	36	398	30	190	RPV/TAF/FTC	<40	175,000	2.98	08:01:01G + 13:02
2781	42	M	Caucasian	2009	433	27	714	44	267	ABC/TCV/3TC	<40	187,090	2.2	N/A
7705	63	M	African American	1987	594	39	531	35	300	ATV, RTV, FTC/TAF	<40	N/A	10	N/A
7726	56	M	Caucasian	1986	679	32	938	45	235	BIC/FTC/TAF	<40	N/A	8.36	N/A

^1^ Abbreviations: VL = viral load; 3TC = lamivudine; ABC = abacavir; ATV = atazanavir; BIC = bictegravir sodium; COBI = cobicistat; DRV = darunavir; DTG = dolutegravir; EFV = efavirenz; EVG = elvitegravir; FTC = emtricitabine; MVC = maraviroc; RAL = raltegravir; RPV = rilpivirine; RTV = ritonavir; TAF = tenofovir alafenamide; TCV = tivicay; TDF = tenofovir. ^2^ N/A = not available. ^3^ Protective HLA alleles (green) and deleterious HLA alleles (red) are indicated.

## Data Availability

Data are contained within the article or Supplementary Materials.

## Supplementary Materials

The following supporting information can be downloaded at https://www.mdpi.com/article/10.3390/v15071606/s1. Figure S1: Probe optimization comparison for ddPCR and dPCR; Figure S2: Levels of intact versus total HIV DNA in cohort; Figure S3: HIV transcription levels relative to reservoir size; Figure S4: Correlation between years of HIV suppression under ART and HIV transcripts.
