# Supplementary material for: Adaptation of Droplet Digital PCR-Based HIV Transcription Profiling to Digital PCR and Association of HIV Transcription and Total or Intact HIV DNA
Source: Viruses. 2023 Jul 22;15(7):1606. doi: 10.3390/v15071606 (PMC10384802; doi:10.3390/v15071606)
Supplement: Supplementary file 1 [file viruses-15-01606-s001.zip › viruses-2503868-supplementary.pdf]

Supplementary material

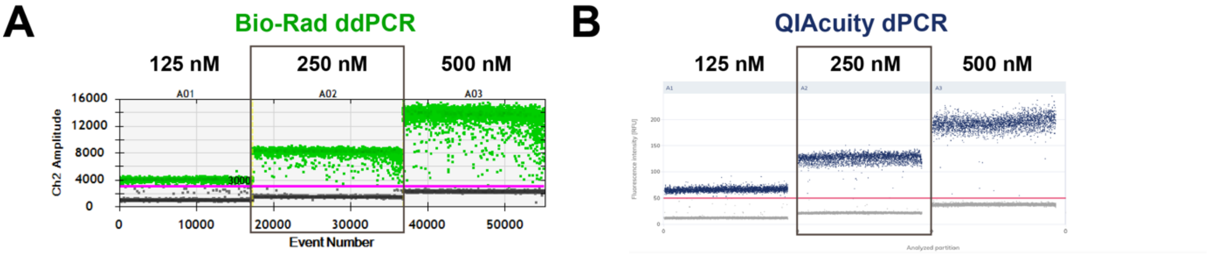

Fig. S1. Probe optimization comparison for ddPCR and dPCR. A series of three probe concentrations were tested (125nM, 250nM, and 500nM) to determine the optimal concentration for assays in the dPCR system. Representative plots for Pol assay are shown. The probe concentration selected for further studies (250nM) is shown (grey box).

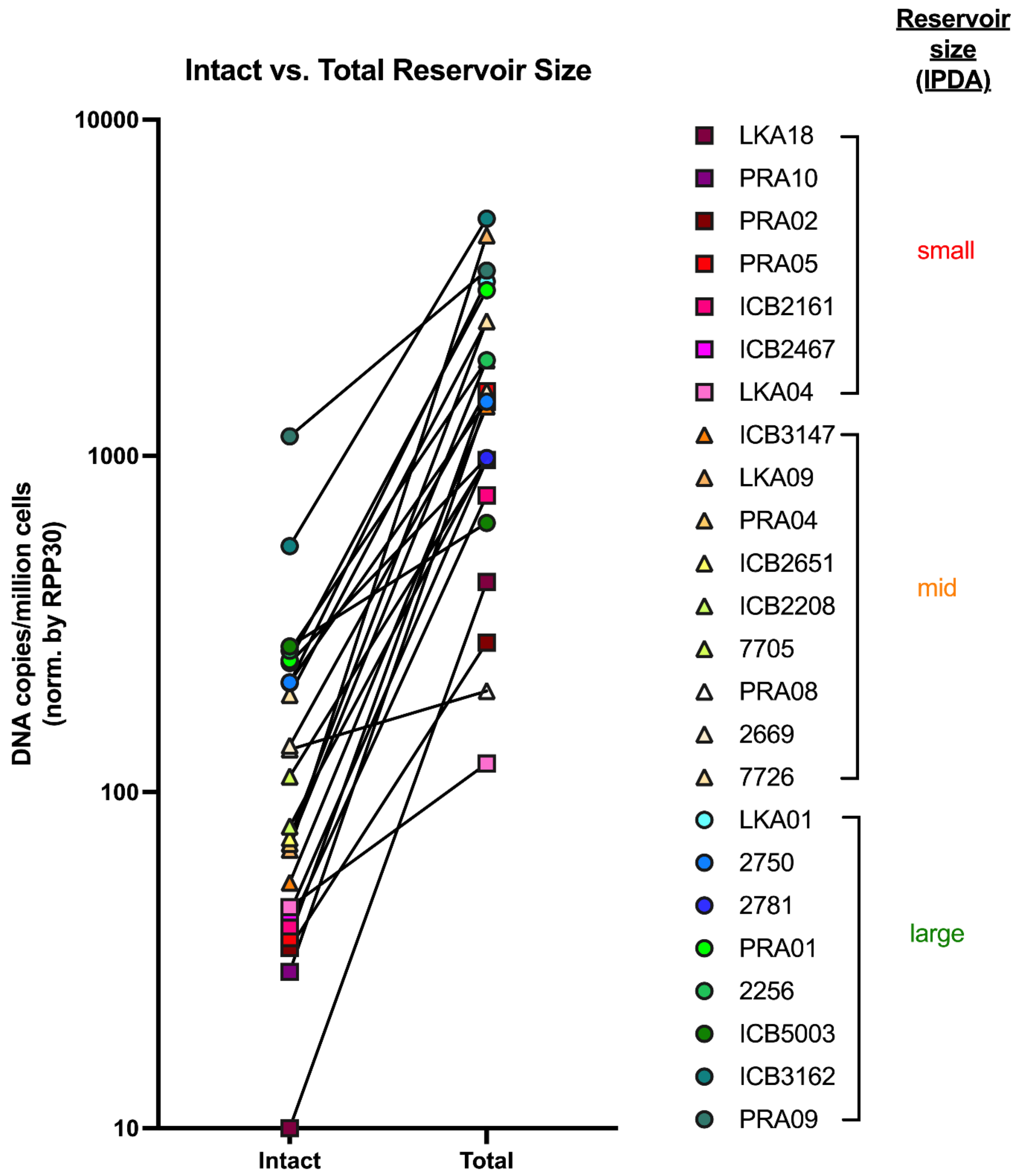

Fig S2. Levels of intact versus total HIV DNA in cohort.
Intact and total HIV DNA was measured using the Intact Proviral DNA Asssay (IPDA) and expressed as copies per million cells, normalised by housekeeping gene, RPP30.

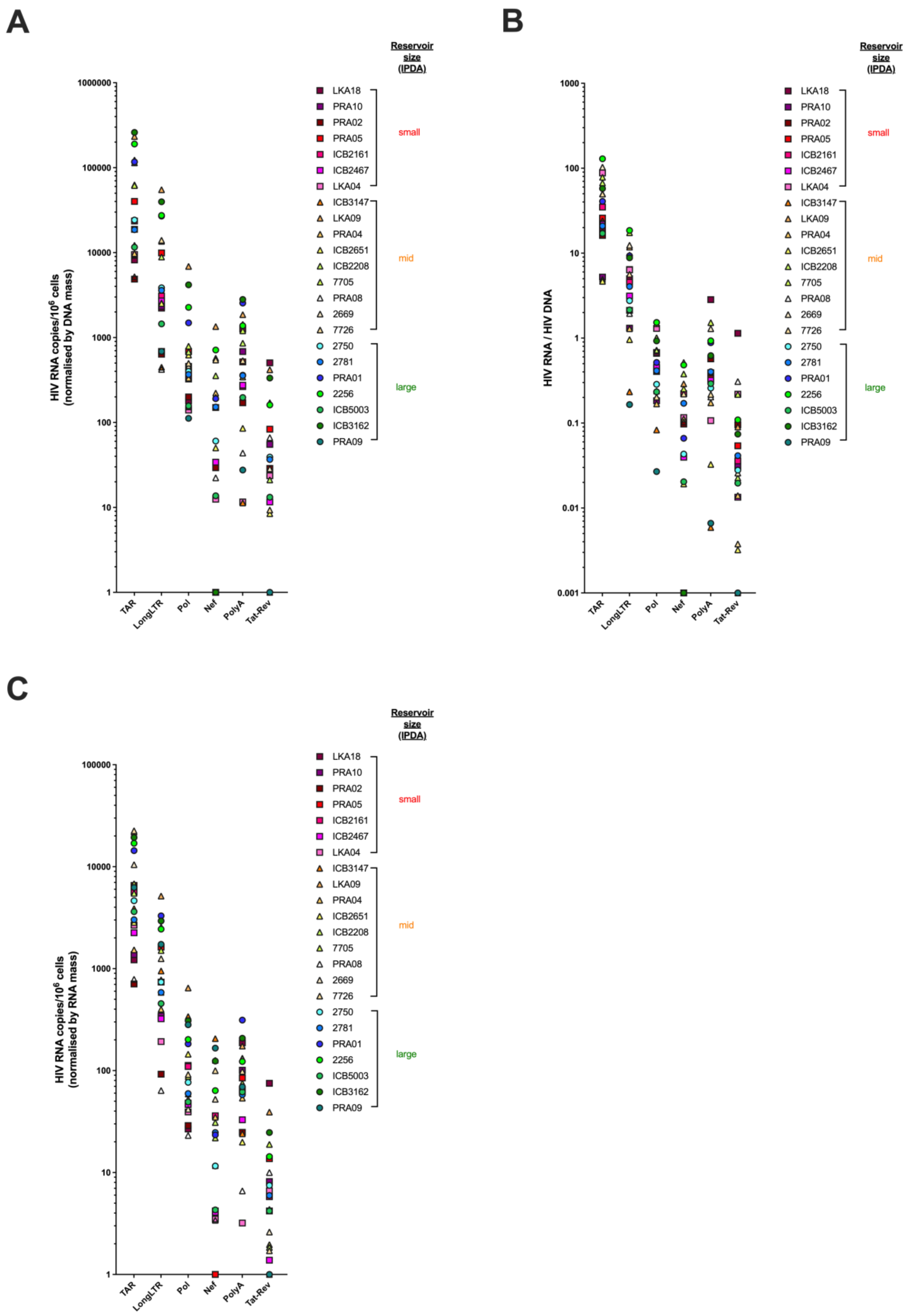

Fig S3. HIV transcription levels relative to reservoir size. HIV transcription profile **(A)** normalised by DNA mass, **(B)** expressed as ratio of HIV RNA per provirus, based on total DNA, and **(C)** normalised by RNA mass. All samples are stratified based on levels of 'intact' proviral DNA.

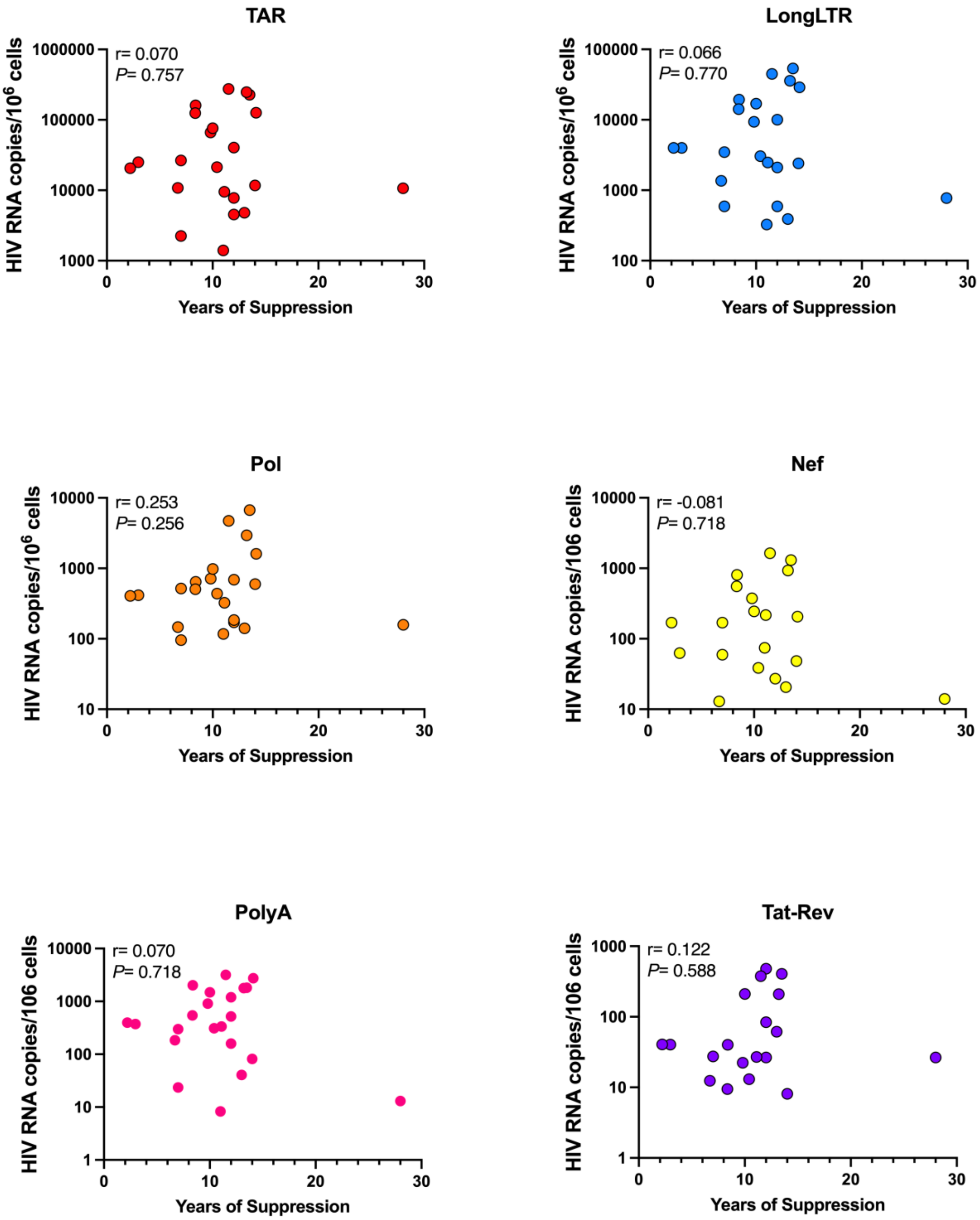

Fig. S4. Correlation between years of HIV suppression under ART and HIV transcripts. HIV RNA copies per million CD4+ T cells normalised by RPP30 for each transcript species is shown. Spearman correlation (r) between years of suppression under ART and HIV transcripts. *P*= p-value.
